# Changes in Behavior After Vaccination and Opinions Toward Mask Wearing: Thoracic Oncology Patient–Reported Experiences During the COVID-19 Pandemic

**DOI:** 10.1177/11795549221123618

**Published:** 2022-09-27

**Authors:** Toki Bolt, Amanda Tufman, Laura Sellmer, Kathrin Kahnert, Pontus Mertsch, Julia Kovács, Diego Kauffmann-Guerrero, Dieter Munker, Farkhad Manapov, Christian Schneider, Juergen Behr, Julia Walter

**Affiliations:** 1 Department of Medicine V, University Hospital, LMU Munich, München, Germany; 2 Comprehensive Pneumology Center Munich (CPC-M), German Center for Lung Research (DZL), München, Germany; 3 Department of Thoracic Surgery, Thoracic Oncology Centre Munich, Ludwig-Maximilian University of Munich, München, Germany; 4 Department of Radiation Oncology, Thoracic Oncology Centre Munich, Ludwig-Maximilian University of Munich, München, Germany

**Keywords:** Corona virus, SARS-CoV-2, thoracic malignancies, social distancing, quarantine

## Abstract

**Background:**

The COVID-19 vaccines, face masks, and social distancing are effective interventions to prevent SARS-CoV-2 infections. In this study, we aimed to determine lung cancer patients’ attitudes toward vaccination, changes in behavior after vaccination, and willingness to continue mask wearing after the pandemic.

**Methods:**

We sent out questionnaires to 220 thoracic oncology patients treated at our lung cancer center in May 2021. The questionnaire focused on patients’ vaccination status, self-reported experiences surrounding vaccination, and assessed changes in behaviors before and after vaccination as well as opinions toward mask wearing after the pandemic. Results are presented as absolute and relative frequencies and means with standard deviation and compared using *t* test, paired *t* test, and analysis of variance test as well as chi^2^ test, and Fisher exact text.

**Results:**

About 91.0% of patients reported having received at least 1 vaccination. About 73.3% of patients reported having at least 1 reaction to the vaccination. The most common reactions were pain at the injection site, fatigue, and headache. After vaccination, patients increased contact with family and friends, use of public transport, and grocery shopping. Overall, the level of willingness to wear masks beyond the end of the pandemic differed according to vaccination status.

**Conclusions:**

Acceptance of the COVID-19 vaccination among thoracic oncology patients in Germany was high. Overall, patients with thoracic malignancies tolerated the COVID-19 vaccination well. Rate of adverse reaction was not higher compared with the general population. After the vaccination, patients increased social contacts and usage of public transport. These changes suggest positive psychological effects on quality of life. While reducing social distancing can increase the risk of infection, our results indicate that an extension of mask mandates after the pandemic would likely be accepted by a majority of thoracic oncology patients, suggesting that our cohort was still aware and in support of other measure of protection.

## Background

The spread of SARS-CoV-2 has not only lead to over 100 000 deaths in Germany,^1^ but also to increased morbidity in other acute and chronic illnesses due to avoidance of seeking medical care,^2^ and increased rates of mental illness such as depression and anxiety during lockdowns.^3,4^ Furthermore, a rise in other diseases such as obesity, type 2 diabetes, and hypertriglyceridemia due to physical inactivity have been noted.^5^

Factors associated with a high risk for severe complications from COVID-19 have been found to be old age, male gender, underlying comorbidities such as hypertension, diabetes, obesity, chronic lung diseases, heart, liver, and kidney diseases, tumors, clinically apparent immunodeficiencies, and local immunodeficiencies.^6,7^ Patients with thoracic malignancies often have more than one of these risk factors, due to their underlying condition itself as well as immunosuppression due to therapy and supportive medications such as corticosteroids. In addition, median age of lung cancer patients at diagnosis was around 69 years in females and 70 in male in Germany.^8^ Dai et al^9^ showed that patients with cancer had higher mortality rates, higher risks for intensive care unit (ICU) admission, higher rates of experiencing at least 1 severe symptom, and a higher risk to need mechanical ventilation compared with healthy controls when infected with SARS-Cov-2. A study of Canadian and US-American former and current tumor patients found that active cancer was significantly associated with 30-day mortality after infection.^10^ In a study of patients with autoimmune hepatitis, the authors found that patients with COVID-19 symptoms reported increased fatigue, anxiety, and itch compared with those without symptoms of COVID-19.^11^ Furthermore, frequent contacts with the health care system lead to a higher risk of infection.^12^

Masks along with social distancing are effective non-pharmaceutical public health interventions to reduce the rate of infection.^13^ However, face masks are not always comfortable to wear due to breathing discomfort^14^; this especially applies to patients with a thoracic malignancy.^15^ Besides reducing the spread of SARS-CoV-2, masks and social distancing were associated with a reduction of other airborne diseases like the common cold, bronchitis, and influenza,^16,17^ which are also potential sources of morbidity and mortality for thoracic oncology patients. The new chronic obstructive pulmonary disease (COPD) guidelines (GOLD guideline 2022) already include a recommendation to wear face masks for exacerbation prevention.^18^ With the introduction of the new mRNA COVID-19 vaccines by the end of 2020, a powerful tool was added to non-pharmaceutical interventions. The introduction of COVID-19 vaccines has significantly reduced the risk of developing severe complications from COVID-19 as well as the rate of hospitalization and death.^19^ So far 75.1% of the German population is fully vaccinated.^20^

Overall, initial studies show mRNA-based SARS-CoV-2 vaccines to be well tolerated with few severe side effects.^13^ So far there is no evidence that patients with cancer show a different toxicity profile compared with the general population. In addition, patients under immune checkpoint inhibitor therapy also did not have an increased risk of immune-related adverse events after receiving an influenza vaccination.^21^

Nevertheless, fear of side effects may prevent individuals from getting vaccinated. As such, the perceived burden of vaccination may be important to patient willingness to receive future booster vaccinations.

Changes in behavior following vaccination are also of importance. A study from the United Kingdom suggests that individuals do not substantially decrease compliance with public health measures such as use of masks, social distancing, and reduced household mixing following vaccination. Especially those with more significant health risks showed higher compliance levels to social distancing measures.^14^ However, other studies found that vaccinated people increased their social contacts after vaccination and decreased other measures like mask wearing and careful hand washing.^22,23^ Consequently, a preprint from Denmark found an increase in infections of 40% in the first 2 weeks after vaccination with Pfizer-BioNTech.^24^ It is unclear whether patterns of behavioral change in thoracic oncology patients are similar to the general population, given their high risk of COVID-19 complications. In the beginning of the pandemic in 2020, we saw that lung cancer patients did limit their social interactions.^15^ In addition, a survey of patients with autoimmune hepatitis who are also vulnerable to infection and severe complications due to a suppressed immune system found that a majority of patients would make changes to their behavior like limiting entertainment outside the home, mask wearing, and limiting interactions with family and friends after the strict stay-at-home orders were relaxed.^11^ However, these surveys were completed before vaccinations were available.

In light of these issues, our study aimed^1^ to determine the vaccination status and self-reported experiences surrounding vaccination in patients with a thoracic malignancy,^2^ to assess changes in behavior before and after vaccination, and^3^ to survey patients’ willingness to continue wearing masks in some settings after the pandemic to reduce the risk of other respiratory infections.

## Methods

### Study design, patient cohort, and data collection

In this cross-sectional study, we surveyed patients with a thoracic malignancy during the COVID-19 pandemic. We included all ambulatory patients seen at our thoracic oncology center between 2018 and end of April 2021. We sent out article-based questionnaires, patient information, and consent forms to the identified patients in mid May 2021. Patients were asked to complete the questionnaire before June 30, 2021, and send it back in a pre-paid envelope accompanied by the signed consent form. Our study team including an epidemiologist, a biologist, and a thoracic oncology specialist designed the questionnaire. It was aimed at evaluating patients’ vaccination status and experiences with the vaccination, assess changes in behavior before and after vaccination, and to survey opinions toward mask wearing after the pandemic ended.

### Ethics

Approval for this cross-sectional non-interventional study was obtained from the responsible Ethics Committee (Reference number 20-273). The study was conducted in accordance with the Declaration of Helsinki, Good Clinical Practice guidelines, and local ethical and legal requirements.

### Vaccination status and experiences with vaccination

Patients were asked to indicate their vaccination status regarding SARS-CoV-2, streptococcal pneumonia, and influenza. In addition, we asked about reasons for not getting vaccinated, the type of vaccine they received (BioNTech/Pfizer, Moderna, AstraZeneca, Johnson & Johnson, other), and reported any perceived negative effects from the vaccination. Patients who indicated being currently under intravenous therapy (chemotherapy and/or immunotherapy) or radiotherapy were asked to indicate the number of days between their last therapy and the vaccination.

### Behavioral changes

At the beginning of the questionnaire, patients were asked about their social contacts and activities in public spaces during January and February of 2021 when vaccinations for SARS-CoV-2 were not widely available yet. In the last part of the questionnaire, we asked these same questions again now for the time period after the vaccination. Patients were asked to indicate their agreement to statements about their behavior on a visual analog scale (VAS) from full agreement = 0 to full disagreement = 100. We asked about avoiding meeting family members outside one’s household, avoiding meeting friends, and avoiding doctor visits. In addition, we asked about grocery shopping habits, use of public transport, and going to places where proper social distancing was not possible.

### Opinions toward mask wearing

To assess patients’ opinions toward mask wearing, we asked patients to rate their agreement with statements about mask wearing after the pandemic on a VAS from 0 = full agreement to 100 = full disagreement. The statements covered the willingness to continue to wear a mask after the end of the pandemic in the clinic, at the doctor’s office, in public transport, and in places where proper social distancing is not possible. In addition, we asked patients to indicate their agreement with statements about having doctors and nursing staff wear masks.

### General information

We documented patient demographics and essential clinical information such as age in years (categorized as <60 years, 60-79 years, and 80 years and older), sex, household size, education level according to years of schooling (low ⩽ 9 years of school, medium = 10-11 years of school, high ⩾ 12 years of school), and current therapy (therapy-free interval or follow-up after curative treatment, current intravenous chemo- or immunotherapy, oral therapy with tyrosine kinase inhibitors [TKI], radiotherapy).

### Statistical analysis

All data were pseudonymized prior to analysis. We reported descriptive statistics as absolute and relative frequencies for categorical and ordinal variables and as mean with standard deviation for all metric variables. We used *t* test and analysis of variance test to compare metric variables between male and female and between age categories and vaccination status, respectively. To compare relative frequencies between groups, we used chi^2^ test, and Fisher exact test (n in cell < 6). To compare behavior before and after vaccination, we used paired *t* test. We applied a threshold of α < 0.05 for significance in all analyses.

Data analysis was performed using R Version 4.0.0. Tables and figures were created in Microsoft Excel.

## Results

### Patient population and demographics

We sent out questionnaires to 220 patients asking to participate in our study, of these 111 (50.5%) responded. Mean age of patients was 66.0 years (SD = 9.7), and 48.2% of respondents were female. Education level was evenly distributed (low = 33.6%, medium = 36.4%, high = 30.9%). Current therapy was documented as intravenous therapy (chemotherapy and/or immunotherapy) for 27.3% of patients (n = 30), radiotherapy for 3.6% (n = 4), oral therapy (tyrosine kinase inhibitors) for 16.4% (n = 18), and no therapy (follow-up after systemic or local therapy) for 53.6% patients (n = 59). Mean household size was 2.1 (SD = 0.9). Table 1 displays all patient characteristics stratified by sex and age category.

**Table 1.** Patient characteristics stratified by sex and age category.

	All patients (n = 111)		Male (n = 58)		Female (n = 53)		*P* value	<60 (n = 27)		60-79 (n = 46)		80 and older (n = 38)		*P* value
	Mean	SD	Mean	SD	Mean	SD		Mean	SD	Mean	SD	Mean	SD	
Age in years	66.0	9.7	66.5	10.1	65.5	9.2	.56							
Household size	2.1	0.9	2.3	0.9	2.0	1.0	.24	2.7	1.1	2.1	0.9	1.8	0.6	.0003
	n	%	n	%	n	%	*P* value	n	%	n	%	n	%	*P* value
Age category														
<60	27	24.5%	12	21.4%	15	28.3%	.64							
60-79	46	41.8%	25	44.6%	21	39.6%								
80 and older	38	34.5%	21	37.5%	17	32.1%								
Female sex	53	48.2%						15	55.6%	21	45.7%	17	44.7%	.64
Education														
Low	37	33.6%	20	35.7%	17	32.1%	.10	4	14.8%	16	34.8%	17	44.7%	.08
Medium	40	36.4%	16	28.6%	24	45.3%		10	37.0%	17	37.0%	13	34.2%	
High	34	30.9%	22	39.3%	12	22.6%		13	48.1%	13	28.3%	8	21.1%	
Household size														
1	25	22.7%	8	14.3%	17	32.1%	.07	3	11.1%	11	23.9%	13	34.2%	.02
2	57	51.8%	34	60.7%	23	43.4%		10	37.0%	24	52.2%	11	28.9%	
3 or more	28	25.5%	15	26.8%	13	24.5%		12	44.4%	22	47.8%	4	10.5%	
Current therapy														
Intravenous therapy	30	27.3%	20	35.7%	10	18.9%	.15	7	25.9%	14	30.4%	9	23.7%	.09
Radiotherapy	4	3.6%	2	3.6%	2	3.8%		2	7.4%	0	0.0%	2	5.3%	
Oral therapy (eg, TKI)	18	16.4%	6	10.7%	12	22.6%		5	18.5%	11	23.9%	2	5.3%	
No therapy (therapy pause, follow-up)	59	53.6%	30	53.6%	29	54.7%		13	48.1%	21	45.7%	25	65.8%	

Patient characteristics of study population stratified by sex and age category. Means with standard deviation for numeric, and relative and absolute frequencies for categorical variables. Education level was defined as low = no or basic high school degree (Haupt- or Volksschule), medium = intermediate high school degree (Mittlere Reife), and high = advanced high school degree (Abitur). *P* values from chi^2^ and Fisher exact test (n in cell < 6) for categorical and from *t* test for numerical variables.

Abbreviations: SD, standard deviation; TKI, tyrosine kinase inhibitor.

### Vaccination status and infection

At the time of the survey, 91.0% (n = 101) of patients had received at least 1 dose of a COVID-19 vaccine, 62.2% (n = 69) were already fully vaccinated. Ten patients reported not being vaccinated and 1 patient did not report their vaccination status. Vaccination rates for SARS-CoV-2 infections were higher compared with rates of streptococcal infection (45.9%) and influenza (67.6%). Reasons for not being vaccinated yet were prior SARS-CoV-2 infection (n = 2), no appointment available (n = 2), appointment was scheduled in the future (n = 1), inpatient hospital stay (n = 1), no reason given (n = 1), and hesitant about vaccination (n = 3). Of the 3 hesitant patients, only 1 was not vaccinated against streptococcal pneumonia and influenza. One of the other 2 was vaccinated against both; the other was vaccinated against influenza. Nine of the 10 unvaccinated patients were currently not under active tumor treatment, and 1 received intravenous therapy. The majority of patients was vaccinated with the BioNTech/Pfizer vaccine (64.4%), the second most common vaccine given was AstraZeneca (23.3%). In total, 4 patients indicated that they had a SARS-CoV-2 infection, 3 in the unvaccinated, and 1 in the vaccinated group (relative risk = 30.3, 95% confidence interval = 3.5, 264.8). More information about vaccinations additionally stratified by sex and age category can be found in Table 2.

**Table 2.** Information on vaccinations stratified by sex and age category.

	All patients		Male		Female		*P* value	<60 years		60-69 years		70 and older		*P* value
	n	%	n	%	n	%		n	%	n	%	n	%	
SARS-CoV-2 infection	4	3.6%	2	3.4%	2	3.8%	1.00	2	7.4%	2	4.3%	0	0.0%	0.22
Knows someone who died of COVID	15	13.5%	6	10.3%	9	17.0%	0.43	4	14.8%	6	13.0%	5	13.2%	1.00
Streptococcal vaccination	51	45.9%	27	46.6%	24	45.3%	1.00	7	25.9%	20	43.5%	24	63.2%	0.01
Influenza vaccination	75	67.6%	36	62.1%	39	73.6%	0.28	15	55.6%	30	65.2%	30	78.9%	0.13
At least 1 dose of COVID vaccination	101	91.0%	54	93.1%	47	88.7%	0.51	24	88.9%	43	93.5%	34	89.5%	0.76
Not vaccinated	10	9.0%	4	6.9%	6	11.3%		3	11.1%	3	6.5%	4	10.5%	
One dose	32	28.8%	19	32.8%	13	24.5%	0.59	7	25.9%	18	39.1%	7	18.4%	0.31
Fully vaccinated	69	62.2%	35	60.3%	34	64.2%		17	63.0%	25	54.3%	27	71.1%	
Reasons for not being vaccinated														
No appointment yet	2	18.2%	1	25.0%	1	14.3%		0	0.0%	0	0.0%	2	50.0%	
Appointment is coming	1	9.1%	0	0.0%	1	14.3%		0	0.0%	1	33.3%	0	0.0%	
Would like to wait some more	3	27.3%	1	25.0%	2	28.6%		0	0.0%	1	33.3%	2	50.0%	
Fear of vaccination interfering with chemotherapy	1	9.1%	0	0.0%	1	14.3%		1	25.0%	0	0.0%	0	0.0%	
Due to SARS-CoV-2 infection	2	18.2%	0	0.0%	2	28.6%		1	25.0%	0	0.0%	0	0.0%	
Due to inpatient hospital stay/rehabilitation	1	9.1%	1	25.0%	0	0.0%		0	0.0%	1	33.3%	0	0.0%	
Other	1	9.1%	1	25.0%	0	0.0%		1	25.0%	0	0.0%	0	0.0%	
First dose given in														
January	2	2.0%	1	1.9%	1	2.1%		0	0.0%	2	4.7%	0	0.0%	
February	8	7.9%	1	1.9%	7	14.9%		2	8.3%	4	9.3%	2	5.9%	
March	25	24.8%	14	25.9%	11	23.4%		6	25.0%	14	32.6%	5	14.7%	
April	47	46.5%	27	50.0%	20	42.6%		11	45.8%	35	81.4%	1	2.9%	
May	14	13.9%	8	14.8%	6	12.8%		4	16.7%	9	20.9%	1	2.9%	
June	5	5.0%	3	5.6%	2	4.3%		1	4.2%	3	7.0%	1	2.9%	
Vaccine														
AstraZeneca + mRNA	3	3.0%	1	1.9%	2	4.3%		1	4.2%	2	4.7%	0	0.0%	
AstraZeneca	24	23.8%	16	29.6%	8	17.0%		5	20.8%	12	27.9%	7	20.6%	
BioNTech	65	64.4%	34	63.0%	31	66.0%		17	70.8%	24	55.8%	24	70.6%	
Moderna	7	6.9%	3	5.6%	4	8.5%		1	4.2%	3	7.0%	3	8.8%	
Johnson & Johnson	1	1.0%	0	0.0%	1	2.1%		0	0.0%	1	2.3%	0	0.0%	
Other	1	1.0%	0	0.0%	1	2.1%		0	0.0%	1	2.3%	0	0.0%	

Abbreviation: SD, standard deviation.

Information on vaccination status, reasons for not being vaccinated, time of vaccination, and type of vaccination stratified by sex and age category. Relative and absolute frequencies of categorical variables. *P* values from chi^2^ and Fisher exact test (n in cell < 6).

### Perception of negative effects of vaccination

**Figure 1.**
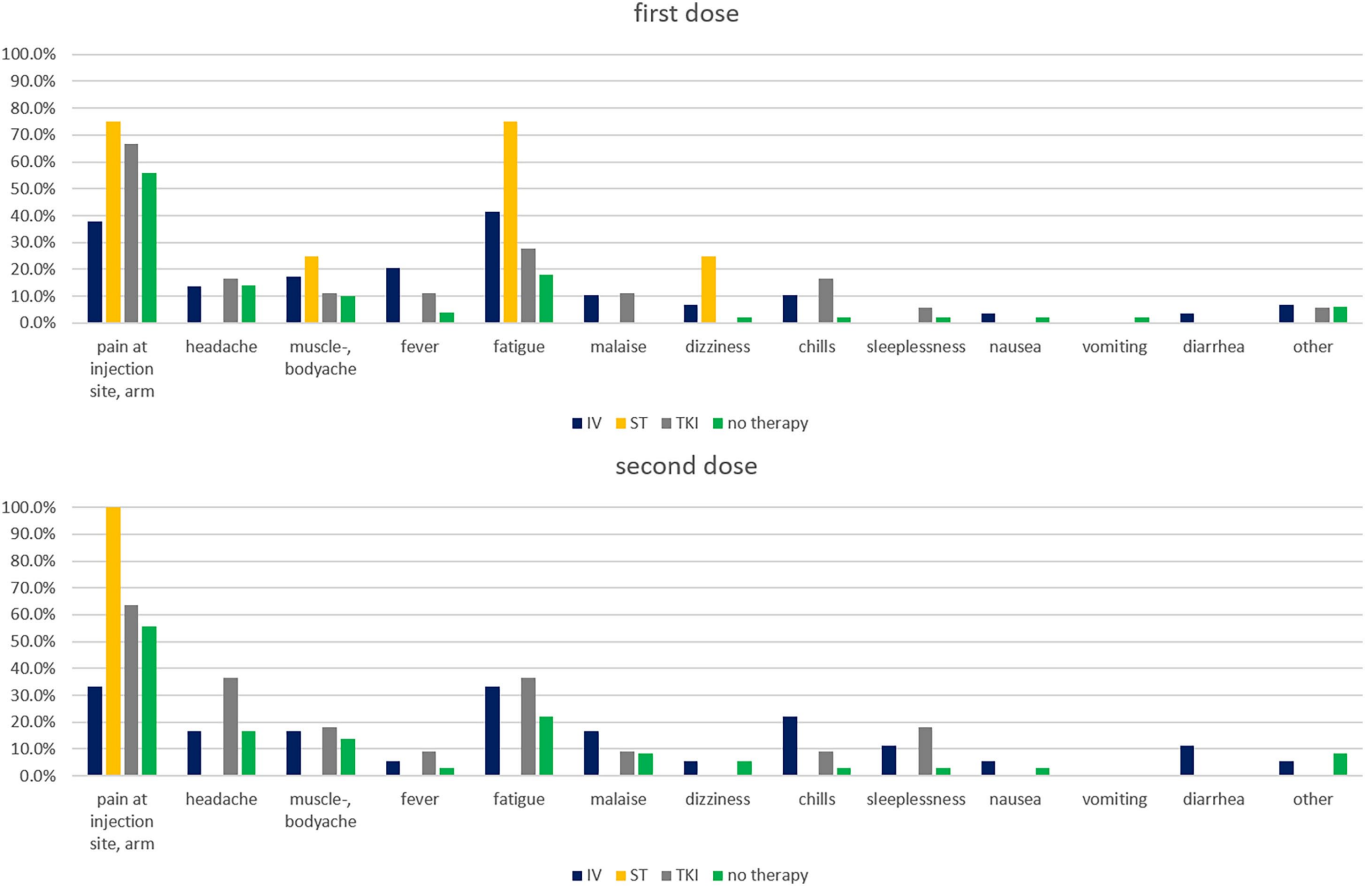
Side effects of first and second dose of vaccination stratified by current therapy. Relative frequencies of self-reported reactions to the first and second dose of the COVID-19 vaccinations, stratified by current therapy. IV indicates intravenous systemic therapy; RT, radiotherapy; TKI, tyrosine kinase inhibitor.

In total, around 73.3% of patients reported having experienced at least 1 negative physical effect following the first and second dose of the vaccine. For the first dose, 19.8% of patients reported having had 3 or more side effects; for the second dose, this proportion was slightly higher (23.5%). The most common side effects reported were pain at the injection site or the arm (first dose 53.5%, second dose 52.9%), fatigue (first dose 28.7%, second dose 26.5%), and headache (first dose 13.9%, second dose 19.1%). Figure 1 shows side effects according to current therapy for the first and second dose. We did not find significant differences in patient-reported side effects across different types of current therapy, neither in specific side effects nor in the number of side effects. Of the patients currently under intravenous or radiotherapy, 33 reported the number of days between their last therapy and the time of vaccination. We did not find a consistent trend regarding the mean number of reported side effects and the time since last therapy. The mean number of days after therapy for patients with no reported side effect was 11.8 (SD = 7.8); for patients with 1 side effect, it was 7.0 (SD = 5.0); for patients with 2, 15.8 (SD = 8.3); and for patients with 3 or more, 22.4 (SD = 23.0).

### Change in social behavior and activities

**Figure 2.**
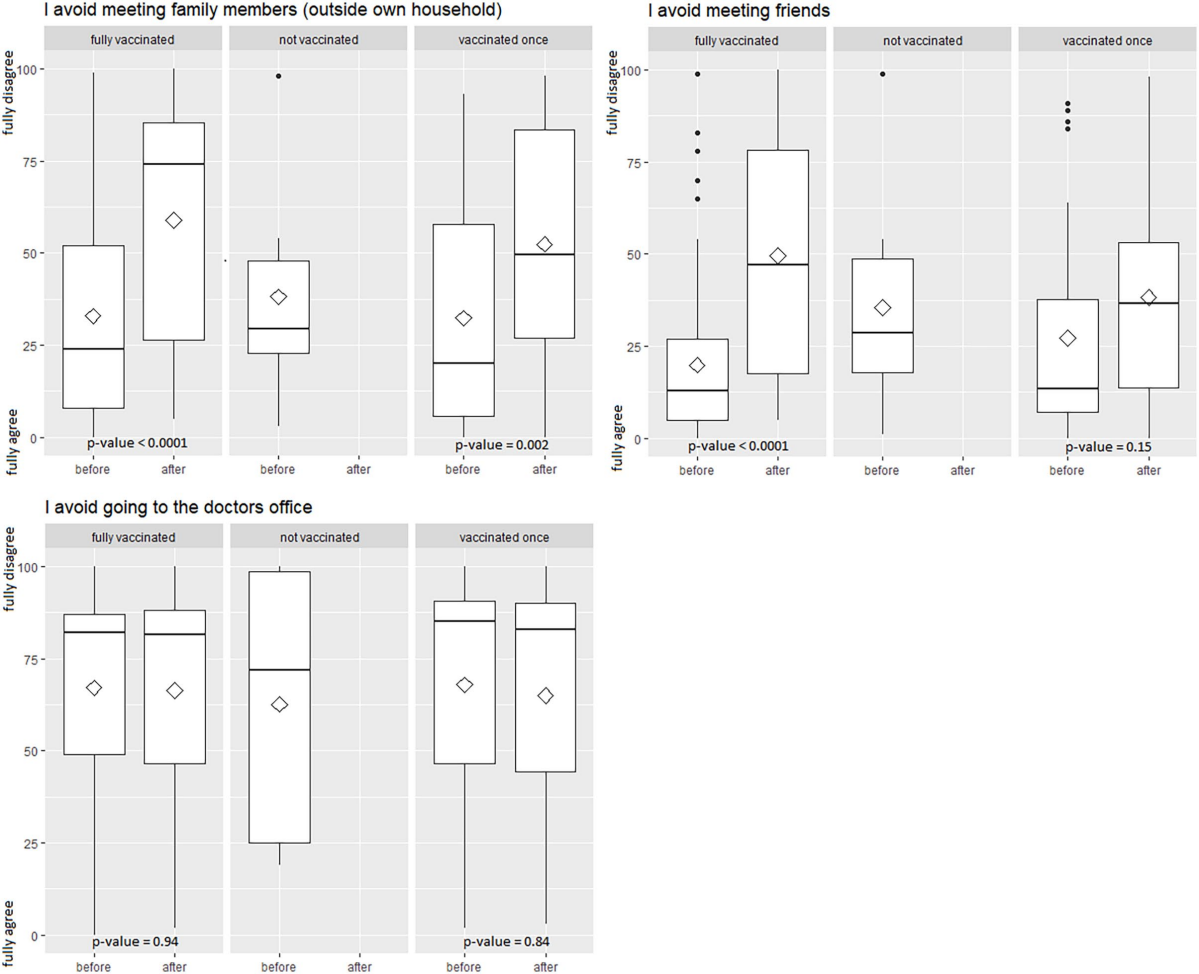
Change in social behavior after vaccination stratified by vaccination status. Boxplots of social behavior before and after COVID-19 vaccinations, stratified by COVID-19 vaccination status. Behavior was measured on a VAS from 0 (full agreement) to 100 (full disagreement) for the time before and the time after vaccination. *P* values are from paired *t* test. VAS indicates visual analog scale.

After being fully vaccinated, patients were more likely to disagree with the statement that they avoided meeting family members (*P* value < .0001), and that they avoided meeting with friends (*P* value < .0001). Patients vaccinated once had a significant shift concerning meeting family member (*P* value = .002), but not concerning meeting friends (*P* value = .15). Hesitancy or likelihood of doctor visits was not affected by vaccination status. Figure 2 shows changes in behavior stratified by vaccination status.

**Figure 3.**
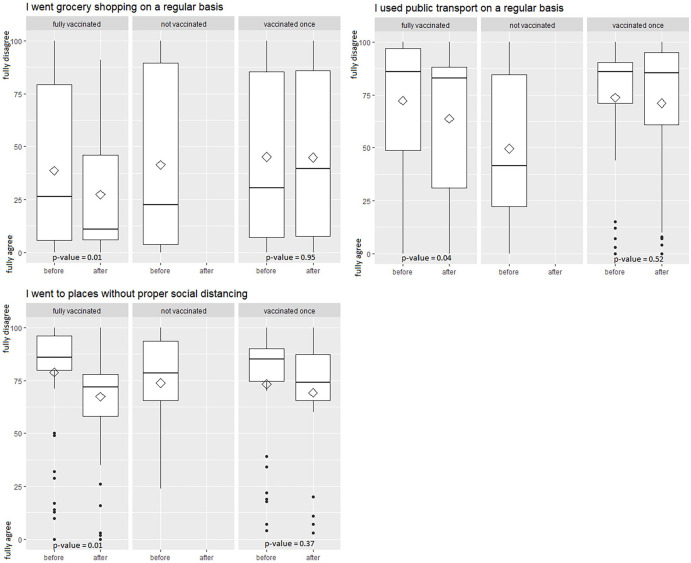
Change in movements after vaccination stratified by vaccination status. Boxplots of movements before and after COVID-19 vaccinations, stratified by COVID-19 vaccination status. Behavior was measured on a VAS from 0 (full agreement) to 100 (full disagreement) for the time before and the time after vaccination. *P* values are from paired *t* test. VAS indicates visual analog scale.

Fully vaccinated patients also had a significant shift in activities like going grocery shopping (*P* value = .009), using public transport (*P* value = .04), and going to places where proper social distancing was not possible (*P* value = .01). Patients with incomplete vaccination status did not change their activities significantly. Figure 3 displays shifts in activities according to vaccination status.

### Opinions toward mask wearing

**Figure 4.**
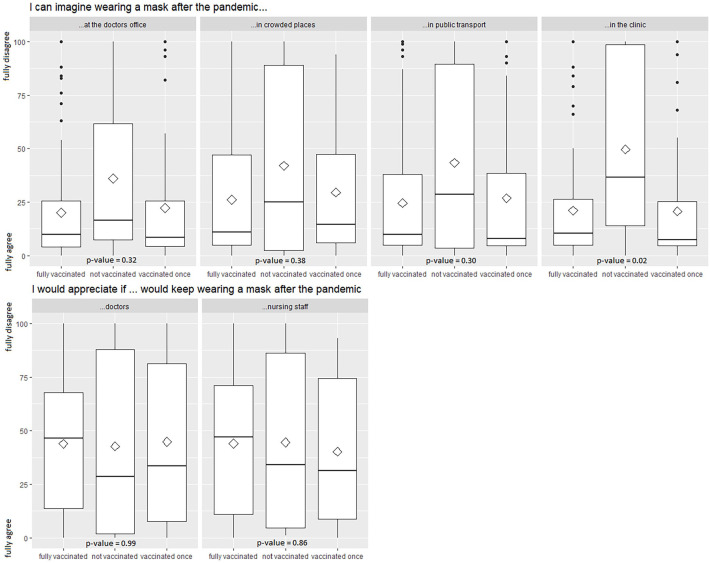
Opinions toward mask wearing after the pandemic according to vaccination status. Boxplots of opinions toward mask wearing, stratified by COVID-19 vaccination status. Opinion was measured on a VAS from 0 (full agreement) to 100 (full disagreement). *P* values are from *t* test. VAS indicates visual analog scale.

On the VAS of 0 to 100 from full agreement to full disagreement, the mean value was 23.1 (SD = 28.8) regarding wearing a mask in the clinic, 21.9 (SD = 28.1) regarding wearing a mask at the doctor’s office, 26.6 (SD = 32.3) regarding public transport, and 28.3 (SD = 30.1) regarding situations without proper social distancing. There was a significant difference in agreement concerning the willingness to wear a mask in the clinic between patients with full vaccination status (*M* = 21.0, SD = 25.7), incomplete vaccination status (*M* = 20.7 SD = 28.4), and no vaccination (*M* = 49.6, SD = 43.3) (*P* value = .02). Complete results can be found in Figure 4.

Opinions toward having doctors and nursing staff wear masks were less affected by vaccination status. We did not find any significant differences here. In general, agreement was lower compared with when asked about wearing a mask themselves. Figure 4 shows these results. An alluvial plot in Figure 1 of the Appendix shows differences in the opinions toward mask wearing depending on age group, sex, and vaccination status.

## Discussion

In our study, 91.0% of patients with a thoracic malignancy reported being vaccinated with at least 1 dose of the COVID-19 vaccine as of the end of June 2021. Only 2.7% of the patients were hesitant to receive the vaccination. Compared with vaccination rates in cancer patients and their reported willingness to be vaccinated in other studies, our patients with a thoracic malignancy demonstrated a high acceptance of the COVID-19 vaccination.^25,26^ Surveys of Polish, Chinese, and Korean cancer patients showed a willingness to be vaccinated of 60.3%,^26^ 46.6%,^25^ and 61.8%,^27^ respectively. Reasons for the high vaccination rate in our study group may, on one hand, be related to the high risk of severe complications from a SARS-CoV-2 infection in patients with a thoracic malignancy and the immunosuppression during therapy. Lung cancer patients had higher mortality rates, higher risks for ICU admission, higher rates of experiencing at least 1 severe symptom, and a higher risk to need mechanical ventilation compared with healthy controls when infected with SARS-CoV-2.^9^ Therefore, their risk-benefit ratio is different compared with healthy people leading to a higher acceptance of the vaccination. On the other hand, patients, especially during active therapy, have a close relationship with the health care system with frequent appointments with clinical oncologists and their primary care physician. This close relationship might have influenced vaccination acceptance, especially as our team promoted an open discussion about the benefits of being vaccinated. This analysis is also supported by the result that 8 of 9 unvaccinated patients were not under active therapy at the time of the survey. Kelkar et al^28^ reported that cancer patients received most of their information about the COVID-19 vaccines from their doctor, the clinic, or the hospital. In addition, Chun et al^27^ found that 91.2% of cancer patients agreed to get vaccinated if their treating physician recommended it. They reported that nearly 30% of patients who were hesitant could be influenced to change their decision depending on their doctor’s recommendation.^27^ In general, when comparing rates of vaccinations, their safety, and efficacy, the timing of the data collection has to be considered. Overall, the good uptake of the COVID-19 vaccination and the perceived association between uptake and promotion of vaccinations by physicians could be used to encourage physicians to further promote vaccinations against other respiratory diseases like influenza and pneumococcal infections.

In general, thoracic oncology patients’ reports of perceived negative effects of COVID-19 vaccination were mild and relatively infrequent compared with the general population. However, 73.3% of patients reported having experienced at least 1 side effect to the first and second dose of the vaccination. The most common side effects reported were pain at the injection site or the arm (first dose 53.5%, second dose 52.9%), fatigue (first dose 28.7%, second dose 26.5%), and headache (first dose 13.9%, second dose 19.1%). Side effects reported in the general public are higher: the Robert Koch Institute reported pain at the injection site in over 80%, fatigue in over 60%, headache in over 50%, muscle pain and chills in over 30%, joint pain in over 20%, and fever and swelling at the injection site in over 10% of patients receiving mRNA vaccines.^13^ Results from a study conducted with cancer patients were more similar to our study. They reported 76.1% adverse events after vaccination, including sore arm (61.7%), fatigue (18.2%), and headaches (12.1%) as the most common events.^29^

In our study, the type of therapy (intravenous chemotherapy and/or immunotherapy or radiotherapy) patients received was not associated with reported negative effects of the vaccination. There was neither an association regarding specific side effects nor the number of side effects after the COVID-19 vaccination. These results are in line with a study in patients treated with a combination of immune checkpoint inhibitors and chemotherapy. The number of adverse events in this study was similar to patient-reported experiences in our study and to healthy controls with the exception of muscle pain which was more present in patients with cancer.^30^ In addition, Luo et al^31^ reported that patients receiving single immune checkpoint inhibitors experienced the typical adverse reactions after COVID-19 vaccination. However, in case of combined immune checkpoint inhibitor therapy (anti-PD-1, anti-PD-L1, anti-CTLA-4), the amount of immune-related adverse events might be increased.^31^ Antibody response after the COVID-19 vaccination was shown to be adequate in a trial comparing the response of the COVID-19 vaccination in patients with a solid tumor receiving chemotherapy, immunotherapy, or chemo-immunotherapy compared with healthy controls.^32^

Regarding social behavior after vaccination, we saw a shift according to the number of injections. Fully vaccinated patients significantly changed their behavior regarding meeting family members, meeting friends and acquaintances, grocery shopping, using public transport, and going to places without proper social distancing. Patients with incomplete vaccination status only significantly changed their behavior toward meeting family members. This phenomenon can be explained by a reduction of perceived risk after the vaccination. After vaccination, our lung cancer patients felt safer in terms of getting infected and regarding severe complications after an infection, especially after they received the second dose. These results are supported by a survey by the UK’s Office for National Statistics which reported that 40% of people indicated that after being vaccinated they would probably follow pandemic-related rules or restrictions less strictly (29%) or not at all (11%).^33^ In addition, a rise in infection rates, probably due to change of behavior before developing immunity against SARS-CoV-2 shortly after vaccination, was reported in England and Israel.^34,35^ However, in general data from the United States suggest lower attack rates and reduced adverse event, ICU hospitalizations, and deaths after infection in the vaccinated.^36^ Nevertheless, fortunately in our study the relative risk of having a SARS-CoV-2 infection was 30.3 [3.5, 264.8] for unvaccinated vs vaccinated patients, indicating that the vaccination, even in immunosuppressed persons and after changed behavior, was effective at the time of the study. In addition, one should not forget positive effects on quality of life, after vaccination. Several studies have found improved psychological conditions and quality of life after vaccination in the general population.^37,38^

Patients did not change the frequency of doctor visits after vaccination. However, most of the patients included in our study were patients with a thoracic malignancy under treatment. Therefore, regular contacts were common before and after vaccination. A survey conducted with participants over 80 years reported they were more likely to seek hospital treatment after 1 injection (25%) and even more after 2 injections (33%). However, we did not find any association between changes in pandemic-related behavior and age.^39^

Overall, there was a significant difference in willingness to wear a mask in the clinic between patients with full vaccination status, incomplete vaccination status, and non-vaccinated patients. Patients refusing to be vaccinated were more likely to object to wearing a mask after the end of the pandemic, which may reflect individual patients’ political views. To our knowledge, this was the first study evaluating the willingness to wear a mask after the end of the pandemic.

This study reports results from a single lung cancer center in Bavaria, Germany, predating the omicron wave. Patients’ experiences and attitudes in other parts of the country might differ due to regional differences during the course of the pandemic (and government restrictions). Questionnaires were mailed out to patients with all types of primary thoracic malignancy and all stages of disease. However, the returned questionnaires might include an element of bias based on patients’ willingness to participate in the survey. Patients with lower symptom burden or acuity of illness might be more willing to answer, and patients with a generally more positive view on vaccination and mask wearing might be more inclined to respond. Also, patients with a regular contact to the clinic might have been more inclined to answer the questionnaire. Another limitation of our study is that we asked patients to recall their behavior from around 4 to 5 months ago. This might introduce some recall bias as well as altruism bias as patients overestimate their ideal expected behavior.^40^ In addition, lung cancer patients especially during active therapy might experience some cognitive impairment, leading to memory failures or making it difficult to fully understand the questions asked in the questionnaire. Furthermore, disease symptoms experienced by patients with lung cancer can differ depending on the histological subtypes. Therefore, all results have to be viewed with this in mind. We sent out our questionnaire in German only; therefore, there might be an underrepresentation of non-native German speakers in our study cohort. Apart from that, baseline patient characteristics were comparable to the general population of thoracic oncology patients. Mean age was 66.7 in our cohort while the mean age at diagnosis of German lung cancer patients in 2016 was 66.0 years in males and 68.3 years in females.^41^ The proportion of females among respondents was 48%, which is a bit higher compared with the proportion of females among newly diagnosed lung cancer patients reported in 2016.^41^ However, lung cancer incidence have been on the rise in females and slightly declining in males in Germany^42^; therefore, a good representation of this emerging cohort can be useful.

Although not all patients responded to our questionnaire, we did receive a response from approximately 50% of patients. This may reflect the high importance of the topic to our patients. In contrast to previous studies, our analysis was specifically focused on behavioral changes of thoracic oncology patients, an especially vulnerable group.

A strength of our study is the prospective nature of our analysis of attitudes toward mask wearing after the pandemic. It is reassuring that most patients are aware that mask wearing is an effective measure to prevent infections that could severely harm them and are willing to continue to use this measure of protection. In analogy to new treatment guidelines for prevention of exacerbations in COPD, we will continue to evaluate the potential benefits of mask wearing for both patients and health care workers preventing all types of respiratory infection.

## Conclusions

Acceptance of the COVID-19 vaccination among thoracic oncology patients in Germany was high. Overall, patients with thoracic malignancies tolerated the COVID-19 vaccination well. Rate of adverse reaction was not higher compared with the general population. Patients reported good compliance with social distancing recommendations, although they also reported changes in their behavior following double vaccination. As the efficacy of 2 doses of the vaccines against the current omicron variants is limited and cancer patients still face severe outcomes,^43^ patients should be cautioned about getting the recommended boosters and practicing social distancing. However, these changes in behavior also suggest positive psychological effects on quality of life, and patients were still supportive of mask wearing even after the pandemic. We believe these results indicate that extending mask mandates in health care settings after the pandemic to avoid other respiratory infections would be supported by a majority of thoracic oncology patients.

## Supplemental Material

sj-docx-1-onc-10.1177_11795549221123618 – Supplemental material for Changes in Behavior After Vaccination and Opinions Toward Mask Wearing: Thoracic Oncology Patient–Reported Experiences During the COVID-19 Pandemic Click here for additional data file. Supplemental material, sj-docx-1-onc-10.1177_11795549221123618 for Changes in Behavior After Vaccination and Opinions Toward Mask Wearing: Thoracic Oncology Patient–Reported Experiences During the COVID-19 Pandemic by Toki Bolt, Amanda Tufman, Laura Sellmer, Kathrin Kahnert, Pontus Mertsch, Julia Kovács, Diego Kauffmann-Guerrero, Dieter Munker, Farkhad Manapov, Christian Schneider, Juergen Behr and Julia Walter in Clinical Medicine Insights: Oncology

sj-png-2-onc-10.1177_11795549221123618 – Supplemental material for Changes in Behavior After Vaccination and Opinions Toward Mask Wearing: Thoracic Oncology Patient–Reported Experiences During the COVID-19 Pandemic Click here for additional data file. Supplemental material, sj-png-2-onc-10.1177_11795549221123618 for Changes in Behavior After Vaccination and Opinions Toward Mask Wearing: Thoracic Oncology Patient–Reported Experiences During the COVID-19 Pandemic by Toki Bolt, Amanda Tufman, Laura Sellmer, Kathrin Kahnert, Pontus Mertsch, Julia Kovács, Diego Kauffmann-Guerrero, Dieter Munker, Farkhad Manapov, Christian Schneider, Juergen Behr and Julia Walter in Clinical Medicine Insights: Oncology
