# Supplementary material for: Changes in Behavior After Vaccination and Opinions Toward Mask Wearing: Thoracic Oncology Patient–Reported Experiences During the COVID-19 Pandemic
Source: Clin Med Insights Oncol. 2022 Sep 27;16:11795549221123618. doi: 10.1177/11795549221123618 (PMC9515761; doi:10.1177/11795549221123618)
Supplement: sj-docx-1-onc-10.1177_11795549221123618 – Supplemental material for Changes in Behavior After Vaccination and Opinions Toward Mask Wearing: Thoracic Oncology Patient–Reported Experiences During the COVID-19 Pandemic [file sj-docx-1-onc-10.1177_11795549221123618.docx]

Written survey about:

Consequences of the COVID-19 pandemic on patients with a chronic lung disease

**Demographics**

1. Please indicate your sex?

☐ female

☐ male

☐ diverse

1. How old are you?

­­­­­­­

_____ years

1. What is you highest school education?

☐ Haupt-/Volksschulabschluss oder gleichwertig

☐ Realschulabschluss oder gleichwertig

☐ Abitur oder gleichwertiger Abschluss

1. What is your highest professional education?

☐ no professional education

☐ beruflich-betriebliche Berufsausbildung (Lehre)

☐ beruflich-schulische Ausbildung (Berufsfachschule, Handelsschule)

☐ Ausbildung an einer Fachschule, Meister-, Technikerschule, Berufs- oder Fachakademie

☐ Fachhochschul-, oder Hochschulabschluss

☐ anderer beruflicher Abschluss, und zwar: _________________________

1. How many persons are currently living in your household including kids?

Number: ______________ persons

**Impact of the pandemic on daily living**

1. Please mark on the line below the point that best describes your daily activities during **January/February 2021**:

Example:

The weather today is good

0 100

fully agree fully disagree

**In January/February 2021:**

| **Due to the risk of infection with COVID-19 I avoided:** |
| --- |
| - Contact with family members (outside my own household).   0 100  fully agree fully disagree |
| - Contact with friends and acquaintances.  0 100  fully agree fully disagree |
| - Contact with doctors.  0 100  fully agree fully disagree |

| **In January/February 2021:** |
| --- |
| - I went grocery shopping regularly.   0 100  fully agree fully disagree |
| - I used public transportation regularly.   0 100  fully agree fully disagree |
| - I went to places where social distancing was not possible.  0 100  fully agree fully disagree |

1. Please mark on the line below the point that best describes you:

| **Even after the pandemic ends:** |
| --- |
| - I could imagine wearing a (FFP-2) mask for appointments in the clinic   0 100  fully agree fully disagree |
| - I could imagine wearing a (FFP-2) mask for appointments at the doctor’s office   0 100  fully agree fully disagree |
| **Even after the pandemic ends:** |
| - I could imagine wearing a (FFP-2) mask in public transport   0 100  fully agree fully disagree |
| - I could imagine wearing a (FFP-2) mask in places where there are a lot of people without social distancing   0 100  fully agree fully disagree |
| - I would be happy if doctors would continue wearing (FFP-2)masks   0 100  fully agree fully disagree |
| - I would be happy if nursing staff would continue wearing (FFP-2)masks   0 100  fully agree fully disagree |

**Coronavirus infection and vaccination**

1. Have you had a confirmed diagnosis with COVID-19? (positive PCR-Test)

☐ no ☐ yes: ______________ (month/year)

1. Which of the following vaccination have you gotten in the last 5 years?

☐ pneumococcal (pneumonia) ☐ influenza (flu)

☐ I don’t know ☐ none of the above

1. Have you been vaccinated against COVID-19?

☐ yes ☐ no

1. If you **haven’t** been vaccinated, what is the reason?

☐ no appointment

☐ appointment has been made

☐ difficulties registering

☐ I would like to wait some more

☐ I don’t want to/can’t get vaccinated. Reason: _________________________

1. When did you get vaccinated (month)?

Fist dose _________ second dose _________

Which vaccine did you get?

☐ AstraZeneca ☐ Biontech ☐ Moderna

☐ Johnsen&Johnsen ☐ other

1. Did you experience on or more of the following reactions?

| Fist dose | second dose |
| --- | --- |
| ☐ pain at the injection site/arm  ☐ headache  ☐ muscle-, body ache  ☐ fever  ☐ fatigue  ☐ malaise  ☐ dizziness  ☐ chills  ☐ sleeplessness  ☐ nausea  ☐ vomiting  ☐ diarrhea  ☐ other: ___________________ | ☐ pain at the injection site/arm  ☐ headache  ☐ muscle-, body ache  ☐ fever  ☐ fatigue  ☐ malaise  ☐ dizziness  ☐ chills  ☐ sleeplessness  ☐ nausea  ☐ vomiting  ☐ diarrhea  ☐ other: ___________________ |

1. Which anti-tumor therapy do you receive currently?

Chemotherapy ☐ yes ☐ no

Immunotherapy ☐ yes ☐ no

Oral therapy (tablets) ☐ yes ☐ no

Radiation therapy ☐ yes ☐ no

Follow-up ☐ yes ☐ no

Therapy pause ☐ yes ☐ no

1. If you are currently under active therapy (Chemotherapy, Immunotherapy, Radiation therapy), what was the timing of the vaccination in relation to the therapy?

| First dose | Second dose |
| --- | --- |
| ____ days after the last and  ____ days prior to the therapy | ____ days after the last and  ____ days prior to the therapy |

1. Please mark on the line below the point that best describes your daily activities during **after being vaccinated**:

| **Due to the risk of infection with COVID-19 I avoid:** |
| --- |
| - Contact with family members (outside my own household).   0 100  fully agree fully disagree |
| - Contact with friends and acquaintances.  0 100  fully agree fully disagree |
| - Contact to doctors.  0 100  fully agree fully disagree |

| **Since being vaccinated:** |
| --- |
| - I go grocery shopping regularly.   0 100  fully agree fully disagree |
| - I use public transportation regularly.   0 100  fully agree fully disagree |
| - I go to places where social distancing is not possible.  0 100  fully agree fully disagree |
