# Supplementary figures and images for: Changes in Behavior After Vaccination and Opinions Toward Mask Wearing: Thoracic Oncology Patient–Reported Experiences During the COVID-19 Pandemic
Source: Clin Med Insights Oncol. 2022 Sep 27;16:11795549221123618. doi: 10.1177/11795549221123618 (PMC9515761; doi:10.1177/11795549221123618)

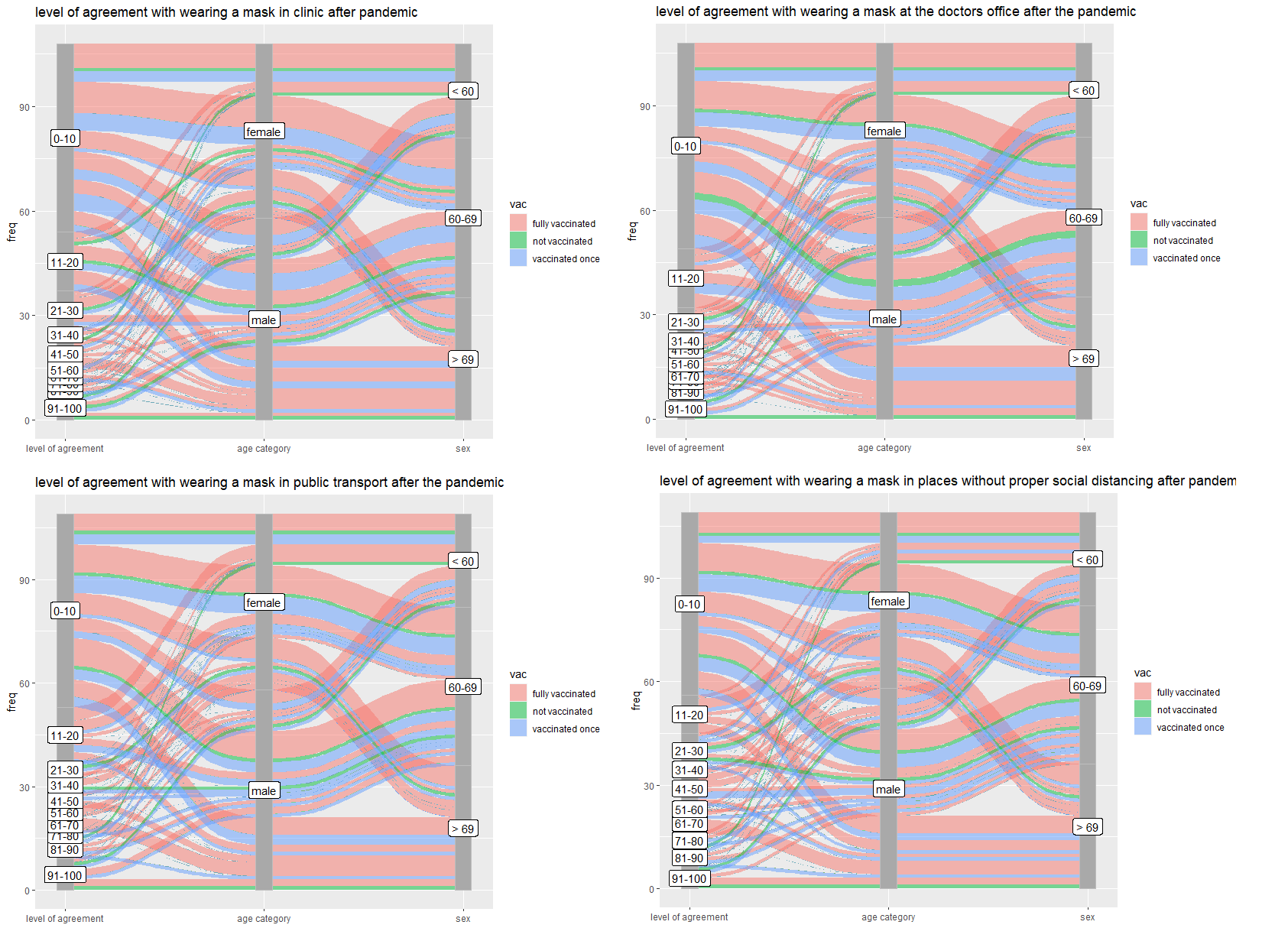

Supplement: sj-png-2-onc-10.1177_11795549221123618 – Supplemental material for Changes in Behavior After Vaccination and Opinions Toward Mask Wearing: Thoracic Oncology Patient–Reported Experiences During the COVID-19 Pandemic [file sj-png-2-onc-10.1177_11795549221123618.png]
